# Exploring the structural, electronic, and hydrogen storage properties of hexagonal boron nitride and carbon nanotubes: insights from single-walled to doped double-walled configurations

**DOI:** 10.1038/s41598-024-55583-8

**Published:** 2024-02-29

**Authors:** Mahmoud A. S. Sakr, Hazem Abdelsalam, Nahed H. Teleb, Omar H. Abd-Elkader, Qinfang Zhang

**Affiliations:** 1 Center of Basic Science (CBS), Misr University for Science and Technology (MUST), 6th October City, Egypt; 2 School of Materials Science and Engineering, Yancheng Institute of Technology, Yancheng, 224051 People’s Republic of China; 3 Theoretical Physics Department, National Research Centre, El-Buhouth Str., Dokki, Giza, 12622 Egypt; 4 Electron Microscope and Thin Films Department, National Research Centre, El-Buhouth Str., Dokki, Giza, 12622 Egypt; 5 Department of Physics and Astronomy, College of Science, King Saud University, P.O. Box 2455, 11451 Riyadh, Saudi Arabia

**Keywords:** Carbon and hexagonal BN Nanotubes, Doping, DFT, TD-DFT, Adsorption energy, Hydrogen storage, Chemistry, Materials science, Physics

## Abstract

This study investigates the structural intricacies and properties of single-walled nanotubes (SWNT) and double-walled nanotubes (DWNT) composed of hexagonal boron nitride (BN) and carbon (C). Doping with various atoms including light elements (B, N, O) and heavy metals (Fe, Co, Cu) is taken into account. The optimized configurations of SWNT and DWNT, along with dopant positions, are explored, with a focus on DWNT-BN-C. The stability analysis, employing binding energies, affirms the favorable formation of nanotube structures, with DWNT-C emerging as the most stable compound. Quantum stability assessments reveal significant intramolecular charge transfer in specific configurations. Electronic properties, including charge distribution, electronegativity, and electrical conductivity, are examined, showcasing the impact of doping. Energy gap values highlight the diverse electronic characteristics of the nanotubes. PDOS analysis provides insights into the contribution of atoms to molecular orbitals. UV–Vis absorption spectra unravel the optical transitions, showcasing the influence of nanotube size, dopant type, and location. Hydrogen storage capabilities are explored, with suitable adsorption energies indicating favorable hydrogen adsorption. The desorption temperatures for hydrogen release vary across configurations, with notable enhancements in specific doped DWNT-C variants, suggesting potential applications in high-temperature hydrogen release. Overall, this comprehensive investigation provides valuable insights into the structural, electronic, optical, and hydrogen storage properties of BN and C nanotubes, laying the foundation for tailored applications in electronics and energy storage.

## Introduction

Addressing the EU's net-zero emission goals involves a strategic shift from fossil fuels to renewable energies. Within the realm of renewable energies, hydrogen stands out as a green fuel. Notably, hydrogen boasts a significantly higher power density compared to other sources such as wind, solar, and ion batteries. However, harnessing hydrogen as an energy source and fuel necessitates a pivotal and challenging step: the development of materials with enhanced storage capabilities. The design of new materials with high storage capacity involves a careful consideration of various factors, including charge/discharge time (efficiency), stability, and sensitivity^1,2^. Achieving advancements in these material properties is critical for optimizing the utilization of hydrogen and aligning with the ambitious goals of achieving a carbon–neutral future in the European Union.

Hexagonal-BN, also known as white graphene, has garnered significant attention in the realm of materials science due to its unique properties^3^. Researchers are particularly interested in exploring the potential of hexagonal-BN as a two-dimensional material. The combination of nanostructured two-dimensional materials (2DMs) gives rise to heterostructures, offering distinctive properties^4^. Several factors contribute to hexagonal-BN being a promising candidate for hydrogen adsorption applications. Its layered structure results in a high surface area, providing ample suitable sites for hydrogen adsorption^5^. The structural similarity to graphene, a well-studied material used in electrochemical hydrogen storage, is another advantageous factor^6^. The chemically stable form of hexagonal-BN prevents contamination, and degradation, and increases the material's lifespan. Its low density is crucial for the development of hydrogen storage applications in utilities like fuel cells and vehicles. Additionally, hexagonal-BN exhibits moderate hydrogen bonding, facilitating the easy adsorption and desorption of hydrogen without the need for excessive temperature or pressure^7^. Despite the abundance of research on graphene and other materials, there is limited literature on the evaluation of hexagonal-BN addition to carbon nanotubes (CNTs). Boateng et al.^8^ reported the effects of adding B nanoparticles to graphene oxide. Combining high thermal conductivity hexagonal-BN particles with high electrical conductivity CNTs to form a CNT/h-BN heterostructure can lead to high-performance electrochemical hydrogen storage. The use of hexagonal-BN as a supportive material improves electron transfer and reduces CNT degradation in electrolytes.

The emergence of nanoscale tubular structures has ushered in a new era of scientific inquiry. Initially synthesized^9^, carbon nanotubes (CNTs) have enthralled researchers with their extraordinary structural, mechanical, chemical, physical, and electronic attributes, paving the way for diverse and innovative applications^10–12^. The behavior of CNTs, whether metallic or semiconductor, hinges on their diameter and chirality^13^. Their noteworthy aspect ratio contributes to substantial electric field enhancement and a low emission threshold voltage^14^. Recent attention has turned towards the synthesis of alternative quasi-one-dimensional nanotubes, particularly those based on other group IV elements, such as silicon carbide nanotubes (SiCNTs). SiCNTs present unique electronic properties, holding promise for the development of nanodevices. In contrast to CNTs, SiCNTs consistently exhibit semiconductor behavior irrespective of their chirality^15^. The capability to fine-tune the electronic structures of semiconducting SiCNTs is crucial for tailoring their applicability in specific nanoscale electronic devices. Similarly, there is growing interest in group III-nitrides, particularly boron nitride nanotubes (BNNTs), owing to their distinctive properties and potential applications. All BNNTs are semiconductive materials with band gaps exceeding 2 eV^16^. Remarkably, BNNTs possess intriguing features, including exceptional resistance to oxidation at elevated temperatures compared to CNTs^17^.

The reported findings highlight that introducing a second particle to carbon-based materials enhances their structural configuration for hydrogen adsorption, leading to improved properties, as demonstrated in various insightful studies^18^. This addition fosters the formation of heterostructures, resulting in a higher specific surface area and, consequently, more sites available for hydrogen adsorption^19^. For instance, Aghajani et al.^20^ incorporated nitrogen heteroatoms into graphene oxide, resulting in a remarkable increase in hydrogen storage capacity. Despite existing studies in the literature detailing the addition of various compounds to carbon-based materials such as graphene oxide (GO) and reduced graphene oxide (rGO)^21^, there is a noticeable scarcity of research exploring the addition of compounds to carbon nanotubes (CNTs)^22^. In summary, our manuscript investigates the structural intricacies, stability, and electronic properties of single-walled (SWNT) and double-walled (DWNT) boron nitride (BN) and carbon (C) nanotubes. We extend our exploration to include dopant atoms, strategically positioned within the nanotube structures, revealing their effects on stability and electronic behavior. The study systematically analyzes binding energies, quantum stability, electronic structure, and optical characteristics. Notably, our investigation into hydrogen storage capabilities presents promising results, especially in doped DWNT-C variants, suggesting potential applications in high-temperature hydrogen release. The comprehensive insights provided in this work contribute to a deeper understanding of these nanotube configurations, offering a foundation for their application in diverse technological fields.

## Computational methodology

This study employs Gaussian 16 simulations based on density functional theory (DFT)^23–30^ to investigate the electrical and optical properties of single-walled (SWNT) and double-walled (DWNT) hexagonal boron nitride (BN) and carbon (C) nanotubes and their dopant forms^31^. The hybrid B3LYP functional^30,32–38^ is used in this work since it has been analyzed and shown to give an adequate representation of the electronic and optical properties of C-based structures^39–42^. The Van der Waals interactions between the layered nanotubes themselves and/or between the nanotubes and the adsorbed molecules are considered by adding Grimme's dispersion correction (gd3) to the B3LYP functional^43^. This correction significantly improves the ability of the B3LYP functional to describe charge transfer and intermolecular interactions.

The 6-31G basis set is chosen due to its accurate results with minimal computational complexity^44^. To analyze the optical properties, time-dependent DFT calculations are performed for the first twenty excited states of the SWNT and DWNT of BN and C and their doped counterparts. The total density of states (TDOS) and partial density of states (PDOS) are analyzed using the Multiwfn 3.7 program^45,46^.

### Ethical approval

This article does not contain any studies involving animals performed by any of the authors.

### Consent to participate

This article does not contain any studies involving animals performed by any of the authors.

## Results and discussion

### Optimized structures

**Figure 1:**
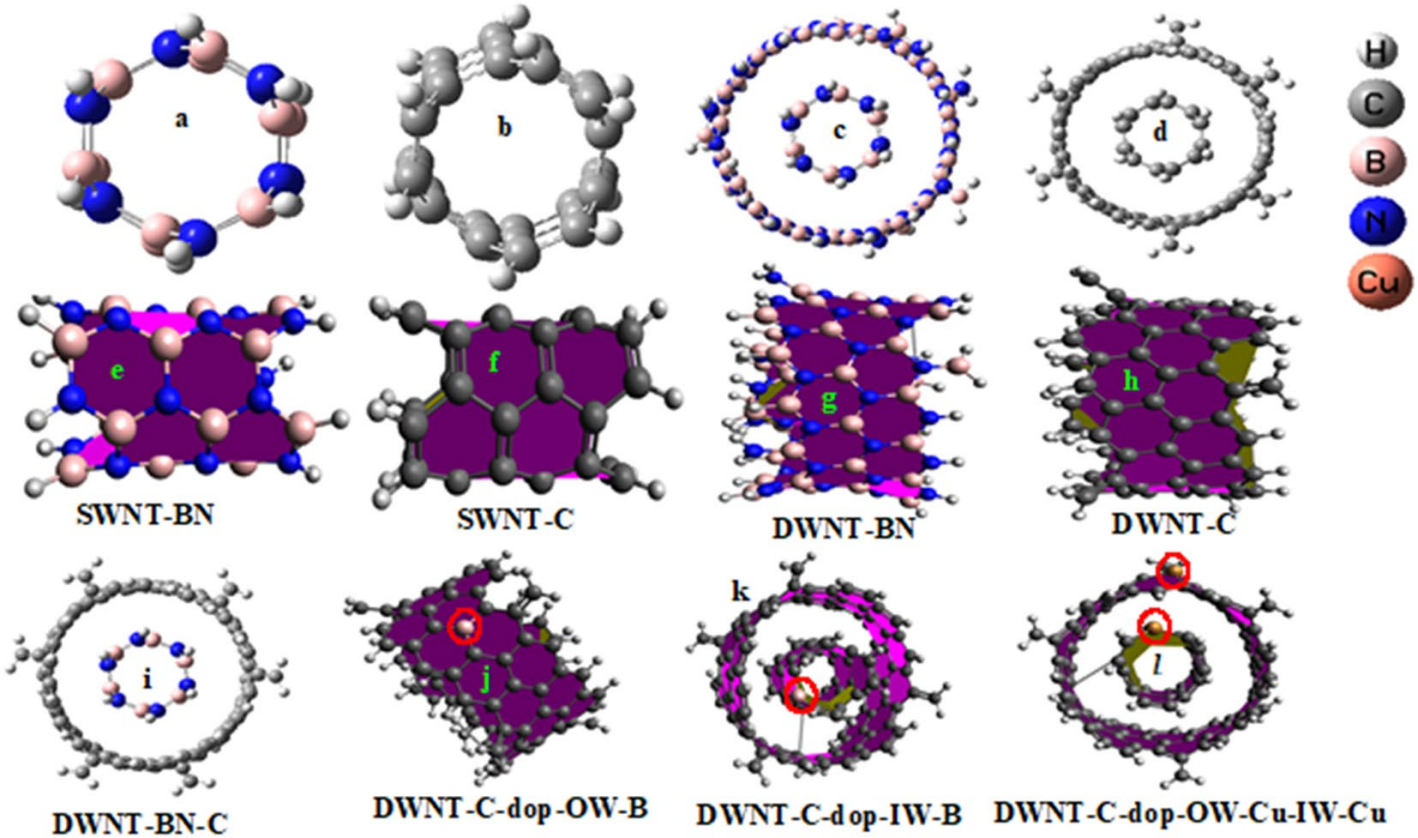
The optimized molecular structures under investigation encompass single-walled nanotubes (SWNT) depicted in figures (**a**, **b**), double-walled nanotubes (DWNT) showcased in figures (**c**, **d**, **i**) as well as the strategic placement of dopant atoms, as illustrated in figures (**j**–**l**). The side view of SWNT and DWNT represented in figures (**e**–**h**).

Our investigation delves into the structural intricacies of both single-walled nanotubes (SWNT) and double-walled nanotubes (DWNT) composed of boron nitride (BN) and carbon (C). For clarity, SWNT and DWNT are used as acronyms throughout this study. Figure 1a–h present optimized front and side-view representations of SWNT and DWNT for both BN and C. Notably, Fig. 1i showcases the optimized DWNT configuration, characterized by an inner wall (IW) of BN and an outer wall (OW) of C, herein referred to as DWNT-BN-C. Furthermore, our investigation extends to the exploration of DWNT-C structures doped with various atoms, including light elements such as B, N, and O, as well as heavier elements like Fe, Co, and Cu. These dopant atoms are strategically positioned within the nanotube structures to assess their effects. Figure 1j,k depict doping positions within the OW and IW, respectively. For instance, Fig. 1j illustrates the introduction of B atoms into the OW, while Fig. 1k showcases B atom doping within the IW. Notably, Fig. 1l offers a comprehensive view of simultaneous doping in both the IW and OW, exemplified by the introduction of Cu atoms into these regions. In sum, this study delves into the structural nuances of SWNT and DWNT composed of BN and C, along with the effects of dopant atom positioning within DWNT-C structures, shedding light on intriguing nanotube configurations with potential applications in various fields.

### Structure stability

In our investigation, we have carefully assessed the stability of BN, C nanotubes, and their related materials by employing the calculation of binding energies (BE). The BE is determined using the following formula: BE = (N_B_E_B_ + N_N_E_N_ + N_C_E_C_ + N_H_E_H_ + N_d_E_d_ − E_t_)/N_t_. Where N_B_, N_N_, N_C_, N_H_, and N_t_ represent the numbers of B, N, C, and H, and the total number of atoms, respectively. In cases involving chemical modifications, N_d_ denotes the number of dopants. E_B_, E_N_, E_C_, E_H_, E_d_, and E_t_ correspond to the total energies of B, N, C, H, dopant atoms, and the final compound, respectively. Our analysis of the calculated binding energies has affirmed the stability of BN, C nanotubes, and related materials. The positive binding energies obtained, ranging from 6.306 to 7.220 eV, signify the favorable formation of these structures. We have tabulated these values for each structure in Table 1. Furthermore, it is worth noting that certain modifications applied to DWNT-C had an impact on the binding energy. The introduction of dopant atoms, including light elements such as O, N, and B, as well as heavy metals like Fe, Cu, and Co, at both the inner wall (IW) and outer wall (OW) resulted in a slight decrease in the binding energy, as outlined in Table 1. Based on the BE values presented in Table 1, it is evident that DWNT-C stands out as the most stable compound. Consequently, we chose this compound as the foundation for further investigations involving the doping of various atoms, including O, N, B, Fe, Co, and Cu, across the IW and OW, as well as simultaneously within both walls. This comprehensive approach allows us to gain valuable insights into the effects of different dopants on the stability and properties of DWNT-C and offers promising prospects for applications in diverse fields.

**Table 1:** The HOMO energy (E_H_), the LUMO energy (E_L_), chemical potential (ρ), electronegativity (χ), chemical hardness (η), dipole moment (μ), the electrical conductivity (σ) and the binding energy (BE) for BN, C NTs, and their derivatives.

Compounds	H (eV)	L (eV)	E_g_	ρ (eV)	χ (eV)	Ƞ (eV)	μ (D)	σ (eV)	BE (eV)
SWNT-BN	− 6.347	− 0.231	6.116	− 3.289	3.289	3.058	0.013	3.64 × 10^−104^	6.306
SWNT-C	− 4.680	− 1.956	2.724	− 3.318	3.318	1.362	0.000	8.51 × 10^−47^	7.124
DWNT-BN	− 6.364	− 0.747	5.617	− 3.556	3.556	2.809	3.227	1.00 × 10^−95^	6.356
DWNT-C	− 4.322	− 2.536	1.786	− 3.429	3.429	0.893	0.214	6.22 × 10^−31^	7.220
DWNT-BN-C	− 4.539	− 2.563	1.976	− 3.551	3.551	0.988	0.156	3.80 × 10^−34^	7.005
DWNT-C-dop-OW-B	− 4.351	− 2.546	1.805	− 3.449	3.449	0.903	2.182	2.96 × 10^−31^	7.197
DWNT-C-dop-OW-N	− 3.437	− 2.403	1.034	− 2.920	2.920	0.517	2.042	3.25 × 10^−18^	7.203
DWNT-C-dop-OW-O	− 3.761	− 2.588	1.173	− 3.175	3.175	0.587	1.374	1.45 × 10^−20^	7.181
DWNT-C-dop-OW-Co	− 4.311	− 2.504	1.807	− 3.408	3.408	0.904	2.103	2.74 × 10^−31^	7.180
DWNT-C-dop-OW-Cu	− 4.307	− 2.622	1.685	− 3.465	3.465	0.843	1.242	3.17 × 10^−29^	7.164
DWNT-C-dop-OW-Fe	− 4.244	− 2.792	1.452	− 3.518	3.518	0.726	3.239	2.77 × 10^−25^	7.184
DWNT-C-dop-IW-B	− 3.922	− 2.541	1.381	− 3.232	3.232	0.691	0.664	4.40 × 10^−24^	7.203
DWNT-C-dop-IW-N	− 3.503	− 2.534	0.969	− 3.019	3.019	0.485	0.458	4.08 × 10^−17^	7.205
DWNT-C-dop-IW-O	− 3.728	− 2.633	1.095	− 3.181	3.181	0.548	2.953	3.02 × 10^−19^	7.193
DWNT-C-dop-IW-Co	− 3.644	− 2.469	1.175	− 3.057	3.057	0.588	2.542	1.34 × 10^−20^	7.192
DWNT-C-dop-IW-Cu	− 3.839	− 2.639	1.200	− 3.239	3.239	0.600	1.054	5.06 × 10^−21^	7.183
DWNT-C-dop-IW-Fe	− 4.016	− 2.637	1.379	− 3.327	3.327	0.690	1.959	4.75 × 10^−24^	7.199
DWNT-C-dop-OW-Cu-IW-Cu	4.026	3.000	1.026	− 3.513	3.513	0.513	4.745	2.25 × 10^−18^	7.129
DWNT-C-dop-OW-O-IW-O	− 3.595	− 2.647	0.948	− 3.121	3.121	0.474	2.937	9.26 × 10^−17^	7.157
DWNT-C-dop-OW-O-IW-Fe	− 3.909	− 2.421	1.488	− 3.165	3.165	0.744	4.703	6.82 × 10^−26^	7.162

### Quantum stability

It is of significant note that structures displaying higher values of μ exhibit an inherent asymmetry in their electronic charge distribution. Within our examined structures, DWNT-C-dop-OW-Cu-IW-Cu, as indicated in Table 1, exhibited the most substantial μ value. Following closely was DWNT-C-dop-OW-O-IW-Fe. These findings suggest that DWNT-C-dop-OW-Cu-IW-Cu undergoes more pronounced intramolecular charge transfer in comparison to other configurations. Conversely, DWNT-BN exhibited the most negative ρ value amongst all systems, signifying a heightened propensity for electron escape. The elevated electronegativity values for DWNT-BN underscore its strong electron-attracting properties. Notably, DWNT-BN displayed the highest χ value of 3.556 among the structures under investigation. Furthermore, we observed that SWNT-BN possessed the highest chemical hardness (η), indicative of its greater resistance to charge transfer when compared to other structures. In contrast, DWNT-C-dop-OW-O-IW-O exhibited the lowest hardness value, suggesting reduced resistance to charge transfer. Our analysis has revealed that electrical conductivity can be significantly altered through processes such as doping, surface functionalization, and chemical modifications^47–51^. To investigate the electrical conductivity of BN, C NTs, and their derivatives for electrons, we employed the equation $\sigma ={\text{exp}}(\frac{{-E}_{g}}{2KT})$^52^, where σ represents electrical conductivity, k is Boltzmann's constant, T is the thermodynamic temperature, and E_g_ corresponds to the band gap value of different configurations. Smaller E_g_ values at a given temperature result in higher conductivity. The computed electrical conductivity values are thoughtfully summarized in Table 1. Notably, our findings in Table 1 highlight that the electrical conductivity of DWNT-C was notably enhanced through the doping of O atoms in both the inner wall (IW) and outer wall (OW). Remarkably, among all the compounds studied, DWNT-C-dop-OW-O-IW-O exhibited the highest electrical conductivity, whereas SWNT-BN displayed the lowest conductivity. In conclusion, our investigation has provided valuable insights into the electronic properties and electrical conductivity of various nanotube configurations. The observed trends in charge distribution, electronegativity, chemical hardness, and electrical conductivity highlight the potential for tailored electronic applications by manipulating the structure and composition of nanotubes.

The values of the energy gap (E_g_) obtained from the data in Table 1 provide crucial insights into the electronic properties of the examined materials. The energy gap represents the difference between the highest occupied molecular orbital (HOMO) energy (H) and the lowest unoccupied molecular orbital (LUMO) energy (L). It is a fundamental parameter that characterizes the electrical conductivity and optical properties of materials. The E_g_ values extracted from Table 1 provide vital insights into the electronic properties of the investigated materials. SWNT-BN exhibits a substantial E_g_ of 6.116 eV, categorizing it as a wide-bandgap material, typically associated with limited electrical conductivity. In contrast, SWNT-C possesses a significantly smaller E_g_ of 2.724 eV, indicative of a narrow-band gap material with better conducting capabilities. DWNT-BN shares a wide bandgap with SWNT-BN (E_g_ = 5.617 eV), while DWNT-C exhibits a smaller E_g_ of 1.786 eV, suggesting enhanced electrical conductivity. DWNT-BN-C also presents a narrow energy gap (E_g_ = 1.976 eV), highlighting its potential as a good conductor or even a metal. These E_g_ values underscore the diverse electronic characteristics of the nanotube structures, with smaller gaps indicating better conductivity, thus offering a range of options for tailored electronic applications. The introduction of dopant materials, including O, N, B, Co, Cu, and Fe, within the inner wall (IW), outer wall (OW), and simultaneously in both walls of double-walled carbon nanotube DWNT-C has a discernible influence on its energy gap (E_g_) values, as depicted in Table 1. Doping at the outer wall (OW) often leads to a reduction in E_g_, signifying a shift toward improved electrical conductivity, with DWNT-C-dop-OW-O exhibiting the lowest E_g_. Conversely, doping at the inner wall (IW) or simultaneous doping at both inner and outer walls can also influence E_g_ values. For instance, DWNT-C-dop-IW-N and DWNT-C-dop-OW-O-IW-O displayed a notably reduced E_g_, indicating enhanced conductivity. These findings underscore the versatility of doping strategies in tailoring the electronic properties of DWNT-C, offering opportunities for fine-tuning its semiconducting or conducting characteristics for specific applications in nanoelectronics and nanomaterials. Comparing the energy gap (E_g_) values obtained using two different density functionals, screened hybrid density functional (PBEPBE)^53^ and B_3_LYP, for DWNT-BN, reveals a notable discrepancy. With PBEPBE, the calculated Eg is 4.215 eV, while B_3_LYP predicts a substantially higher value of 5.617 eV. This considerable difference underscores the sensitivity of density functional theory (DFT) calculations to the choice of functional, where the selection can significantly influence the predicted properties. It is evident that in this case, the B_3_LYP functional yields a significantly larger energy gap compared to PBEPBE, emphasizing the necessity to carefully consider the choice of functional to ensure an accurate interpretation of DFT results.

### Exploring electronic behavior: MOs, PDOS, and doping effects

**Figure 2:**
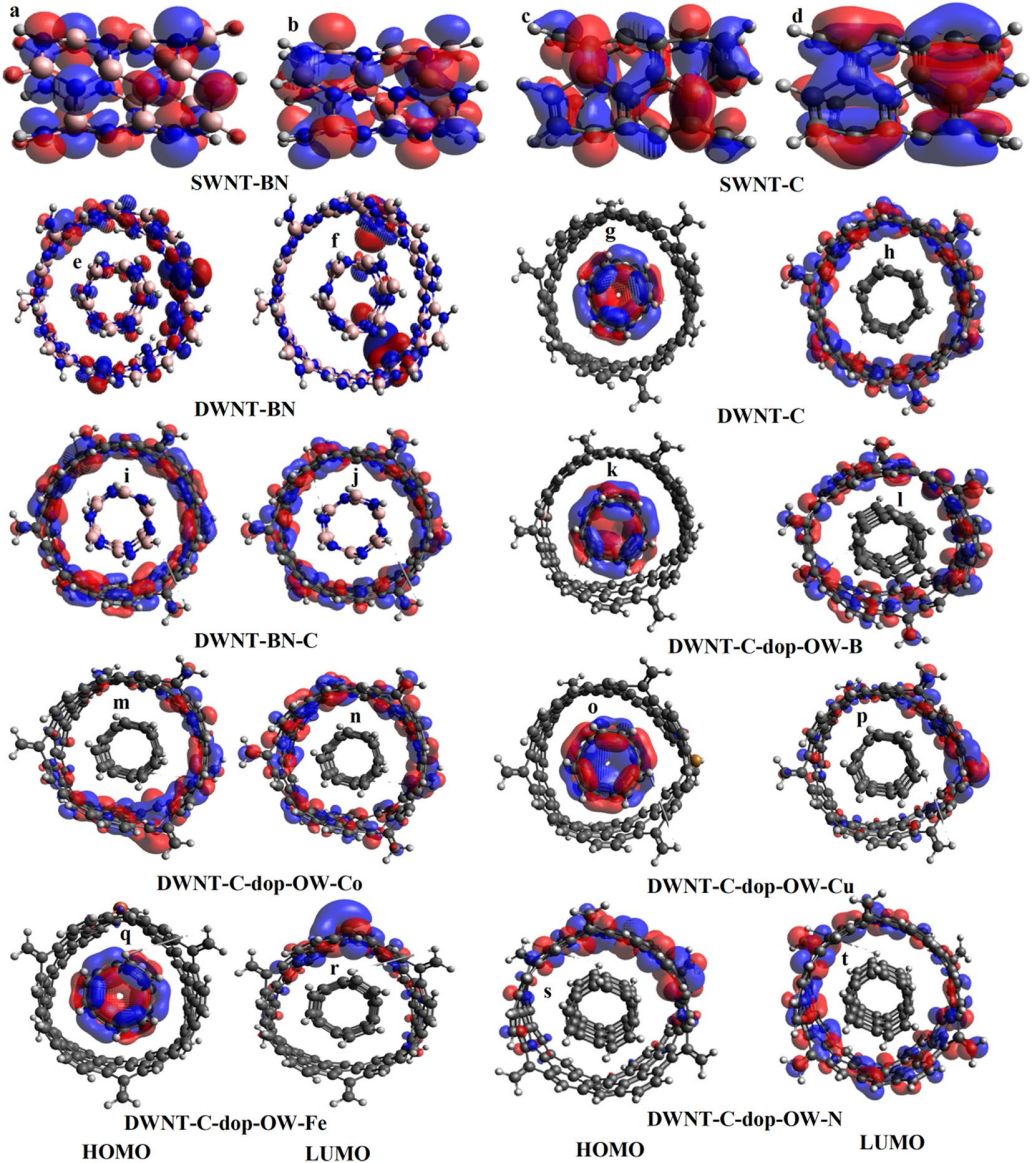
HOMO/LUMO MOs representations for SWNT-BN, SWNT-C, DWNT-BN, DWNT-C, DWNT-C-dop-OW-B, DWNT-C-dop-OW-Co, DWNT-C-dop-OW-Cu, DWNT-C-dop-OW-Fe, and DWNT-C-dop-OW-N (**a**–**t**).

The figures presented in this study provide a comprehensive analysis of the electronic properties and the influence of doping on various nanotube configurations. Figure 2a–t, Figs. S1a–l, and S2e–l depict HOMO/LUMO molecular orbital (MO) representations, offering insights into the spatial distribution of electron density within each nanotube structure, including SWNT-BN, SWNT-C, DWNT-BN, DWNT-C, and doped DWNT-C variants. In Fig. 2a–d, it is observed that in SWNTs, both for BN and C, the HOMO MOs are localized on N atoms and C=C, respectively. This localization is a consequence of the lone pairs of electrons on N atoms and the presence of π bonds between carbon atoms. Conversely, the LUMO MOs are found to be localized on B atoms for SWNT-BN and on C–C=C for SWNT-C. This observation underscores the capacity of B atoms to accept electrons in SWNT-BN and the delocalization of π-bonds between carbon atoms in SWNT-C. Furthermore, in the case of DWNTs, specifically DWNT-BN, the HOMO is localized on N atoms in both the inner wall (IW) and the outer wall (OW), while the LUMO is localized on B atoms in both IW and OW (see Fig. 2e,f). In contrast, for DWNT-C, the HOMO is localized on the carbon atoms of the IW, while the LUMO is localized on the carbon atoms of the OW (see Fig. 2g,h). This distinction highlights the role of carbon atoms in the IW as electron donors and carbon atoms in the OW as electron acceptors. The figures also showcase the HOMO and LUMO MOs of doped DWNT-C variants. Notably, when different atoms are introduced on the IW and OW, or both, in DWNT-C, the doping atoms exert distinctive effects on the distribution of HOMO and LUMO MOs. These effects can be summarized as follows: (1) For DWNT-C variants with doping on IW and OW, HOMO MOs are localized on IW, while LUMO MOs are localized on OW. This applies to cases such as DWNT-C-dop-OW-B, DWNT-C-dop-OW-Cu, DWNT-C-dop-OW-Fe, DWNT-C-dop-IW-Fe, DWNT-C-dop-IW-N, DWNT-C-dop-IW-B, and DWNT-C-dop-IW-Co (Refer to Fig. 2k,l,o–r, Figs. S1a–d, and S2g–j. (2) In certain DWNT-C variants, both HOMO and LUMO MOs are localized on OW. This includes instances like DWNT-C-BN-C, DWNT-C-dop-OW-Co, DWNT-C-dop-OW-N, and DWNT-C-dop-OW-O (Refer to Fig. 2e,f,m,n,s,t, and Fig. S2e,f). (3) In specific cases, HOMO MOs are localized on both IW and OW, while LUMO MOs are confined to IW. This can be observed in DWNT-C-dop-IW-O and DWNT-C-dop-OW-O-IW-O (Refer to Fig. S1*l*,k,h,g). (4) For certain DWNT-C variants, HOMO and LUMO MOs are localized on both IW and OW simultaneously. Notable examples include DWNT-C-dop-OW-Cu-IW-Cu and DWNT-C-dop-OW-O-IW-Fe (Refer to Fig. S1e,f,j,i).

**Figure 3:**
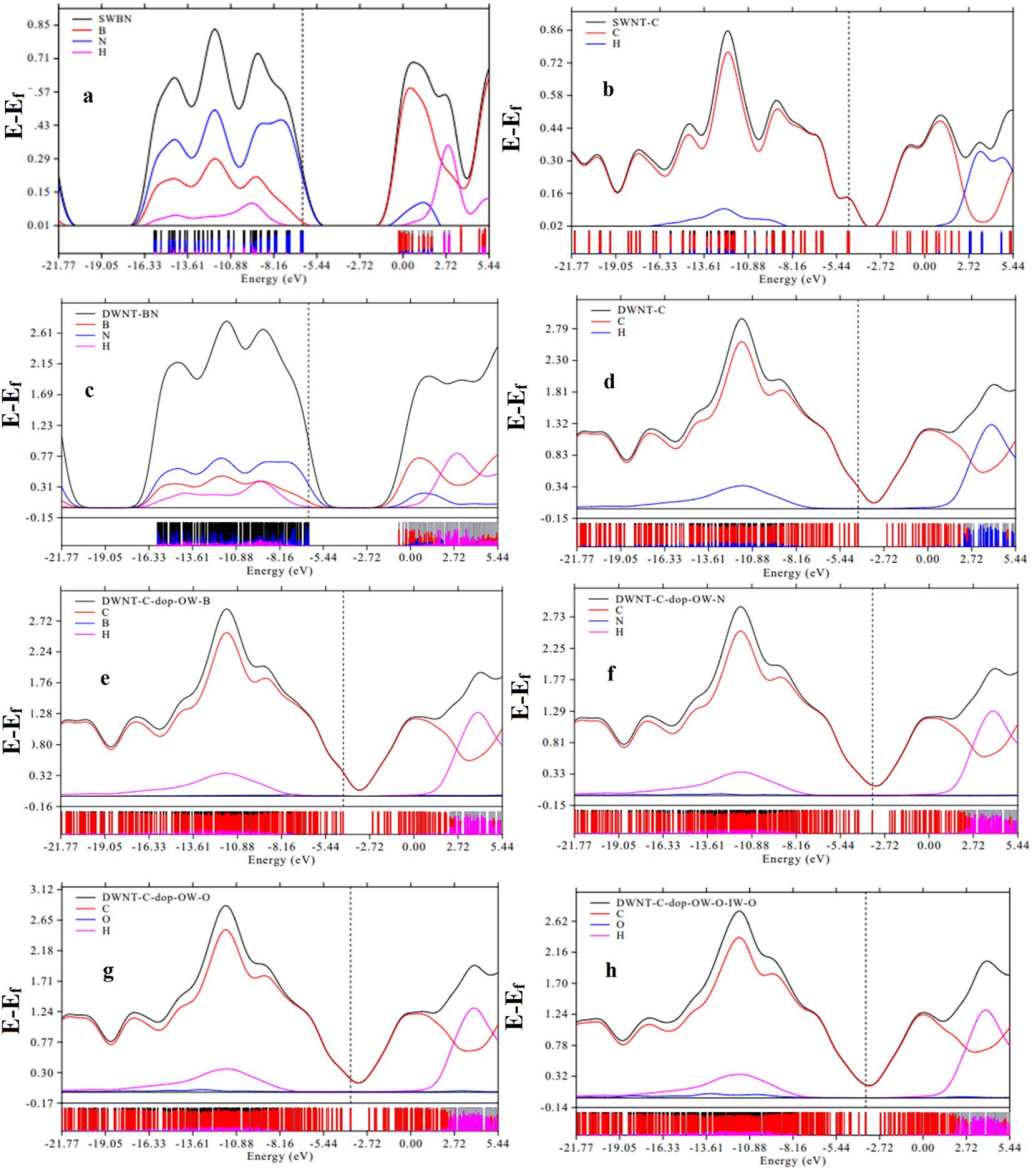
The partial density of states of SWNT-BN (**a**), SWNT-C, DWNT-BN, DWNT-C, DWNT-C-dop-OW-B, DWNT-C-dop-OW-N, DWNT-C-dop-OW-O, DWNT-C-dop-OW-O-IW-O (**b**–**h**). The dotted vertical line is the fermi level.

In our study, we conducted a PDOS (Partial Density of States) analysis using the Multiwfn software. This analysis allowed us to determine the percentage contribution of each atom to the molecular orbitals. Figure 3a–h, Figs. S2a–d, and S3a–h present both the Total Density of States (TDOS) and PDOS for a range of nanotube configurations, including SWNT-BN, SWNT-C, DWNT-BN, DWNT-C, and various doped DWNT-C variants. These figures serve to elucidate the distribution of electronic states within their respective energy spectra. The information derived from these figures plays a crucial role in supporting the data related to the calculation of energy gaps and the HOMO/LUMO molecular orbitals for all the compounds studied. For instance, Fig. 3h highlights the lowest energy gap (0.948 eV) observed in the case of DWNT-C-dop-OW-O-IW-O. This is attributed to the distinctive PDOS peaks associated with oxygen (O) and carbon (C) atoms. Conversely, Fig. 1a illustrates the highest energy gap for SWNT-BN, with this disparity in energy gap values being explained by the distinctive PDOS peaks associated with nitrogen (N), boron (B), and hydrogen (H) atoms. Furthermore, these figures lend support to our interpretations of the HOMO/LUMO representations. Specifically, they confirm the electron-donor role of nitrogen (N) atoms in BNNTs and the electron-acceptor role of boron (B) atoms. On the other hand, in the case of carbon nanotubes (CNTs) and their doped derivatives, carbon (C) atoms are identified as responsible for donating electrons, while both hydrogen (H) and carbon (C) atoms are found to play roles in accepting electrons. In summary, the PDOS analysis conducted through Multiwfn software, as well as the subsequent interpretation of the results in Fig. 3 and Fig. S2, provide crucial insights into the electronic properties of the studied nanotube configurations. These findings support the calculation of energy gaps and the characterization of HOMO/LUMO molecular orbitals, shedding light on the electron-donor and -acceptor roles of specific atoms in these unique structures.

### Optical investigations

**Figure 4:**
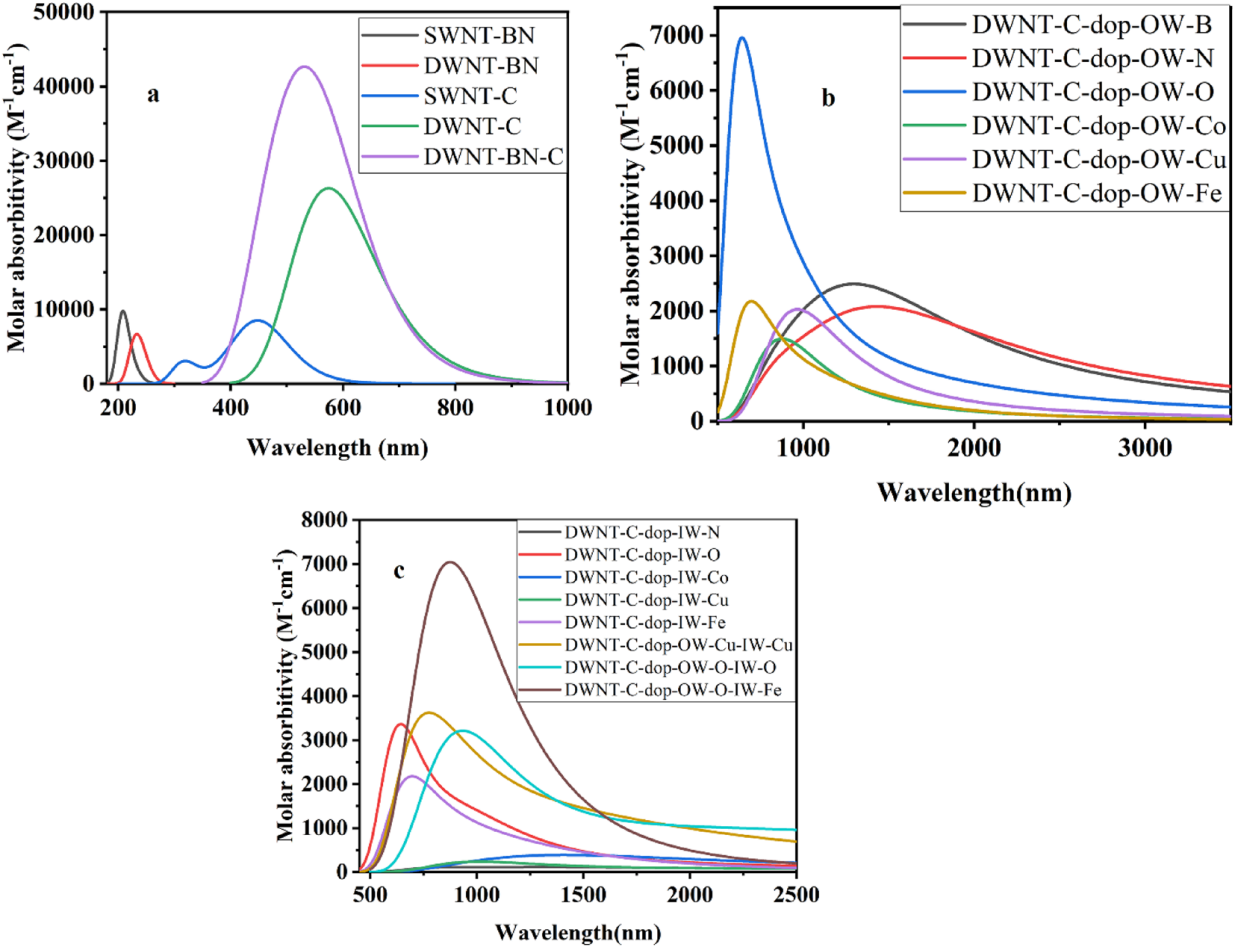
The UV–Vis absorption spectra of SWNT-BN, SWNT-C, DWNT-BN, DWNT-C, and doped DWNT-C variants (**a**–**c**).

The UV–Vis absorption spectra, as depicted in Fig. 4a–c, offer profound insights into the optical characteristics of SWNT-BN, SWNT-C, DWNT-BN, DWNT-C, and various doped DWNT-C variants. These spectra delineate the absorption of photons in relation to their wavelengths, serving as a window into the electronic transitions occurring within the nanotube structures. The corresponding Table 2 provides an exhaustive analysis of the computed optical parameters for all the studied nanotubes. Notably, in the case of SWNT-BN and SWNT-C, we discern distinct excited states (ES) accompanied by maximum wavelengths (λ_max_), transition energies (TE), electronic transition details (ET), oscillator strengths (f), and transition coefficients (TC). For instance, SWNT-BN exhibits an ES of 13, with a λ_max_ of 207.92 nm, indicating a high-energy transition. The TE of 5.963 eV underscores the substantial energy required for this particular electronic transition, originating from the highest occupied molecular orbital (HOMO) to a level 6 below. Conversely, SWNT-C presents an ES of 4, with its absorption spectrum peaking at a significantly longer wavelength (λ_max_ of 455.10 nm) and a TE of 2.1603 eV, suggestive of a lower-energy transition from H − 1 to L + 6. The oscillator strength (f) and transition coefficient (TC) offer further insights into the intensity and nature of these transitions. The transition from individual nanotubes (SWNT-BN and SWNT-C) to double-walled nanotubes (DWNT-BN and DWNT-C) introduces fascinating alterations. DWNT-BN, characterized by an ES of 12, exhibits a blue shift in the absorption spectrum, with a shortened λ_max_ of 233.64 nm, indicative of a higher TE of 5.3065 eV. This shift reflects the modified electronic structure of the double-walled configuration, where the interaction between the inner and outer walls significantly influences the optical characteristics. In contrast, DWNT-C presents 15 ES and experiences a further shift in the absorption spectrum towards longer wavelengths (λ_max_ of 574.28 nm) with a TE of 2.159 eV, signifying electronic transitions from H-2 to L + 1. The introduction of dopants into DWNT-C variants induces a spectrum of changes in the absorption spectra. Notably, the doped DWNT-C structures display λ_max_ values that are notably red-shifted, indicative of lower-energy transitions. The choice of dopant and its specific location within the nanotube structure plays a pivotal role in influencing the shape and peak position of the absorption spectrum. Doping also has the potential to affect both TE and the intensity of electronic transitions, as highlighted by the diverse values of oscillator strengths (f) and transition coefficients (TC). The systematic analysis provided in Table 2 underscores the intricate interplay between nanotube size, dopant type, and their profound influence on the optical properties. These insights bear significance in designing and customizing the absorption characteristics of nanotube-based materials for specific applications, including those in the realm of sensors and optoelectronic devices.

**Table 2:** The calculated excited state (ES), maximum wavelength (λ_max_), transition energy (TE), electronic transition (ET), oscillator strength (*f*), and transition coefficient (TC) for SWNT-BN, SWNT-C, DWNT-BN, DWNT-C, and doped DWNT-C variants.

TD-DFT method						
Compounds	ES	λ_max_ (nm)	TE (eV)	ET	*f*	TC
SWNT-BN	13	207.92	5.963	H → L + 6	0.0978	0.647
SWNT-C	4	455.10	2.1603	H-1 → L + 6	0.1908	0.682
DWNT-BN	12	233.64	5.3065	H → L + 1	0.0450	0.163
DWNT-C	15	574.28	2.159	H-2 → L + 1	0.3202	0.443
DWNT-BN-C	5	516.19	2.1783	H → L + 2	0.0352	0.473
DWNT-C-dop-OW-B	5	1249.86	0.992	H-2 → L	0.0350	0.188
DWNT-C-dop-OW-N	3	1308.51	0.9475	H → L + 3	0.0350	0.868
DWNT-C-dop-OW-O	15	639.44	1.9389	H → L + 7	0.0700	0.485
DWNT-C-dop-OW-Co	13	864.58	1.434	H-3 → L	0.0162	0.614
DWNT-C-dop-OW-Cu	13	938.68	1.3208	H-3 → L + 2	0.0276	0.228
DWNT-C-dop-OW-Fe	14	661.84	1.8733	H → L + 3	0.0400	0.684
DWNT-C-dop-IW-B	12	805.35	1.5395	H → L + 5	0.0080	0.753
DWNT-C-dop-IW-N	3	1412.67	0.8777	H → L + 2	0.0012	0.859
DWNT-C-dop-IW-O	18	635.93	0.47628	H-1 → L + 7	0.0420	2.008
DWNT-C-dop-IW-Co	6	1427.73	0.8684	H → L + 2	0.0034	0.977
DWNT-C-dop-IW-Cu	8	1011.56	1.2257	H → L	0.0012	0.755
DWNT-C-dop-IW-Fe	16	701.23	1.7681	H-5 → L	0.0140	0.344
DWNT-C-dop-OW-Cu-IW-Cu	15	765.65	1.6193	H-5 → L	0.0350	0.639
DWNT-C-dop-OW-O-IW-O	15	927.97	1.3361	H-2 → L	0.0180	0.601
DWNT-C-dop-OW-O-IW-Fe	11	877.54	1.4129	H → L + 2	0.0456	0.402
ZINDO/S method						
DWNT-C	19	523.38	2.3689	H-1 → L + 1	0.712	0.172
DWNT-C-dop-OW-Co	3	1002.85	1.2363	H → L	0.412	0.120
DWNT-C-dop-OW-Cu	1	1238.18	1.0013	H-1 → L	0.0331	0.1063

The ZINDO/S method, as referenced in Refs.^54,55^, is noted for providing superior results in calculating transition energies to higher excited states while also being a very effective and low-cost method. Evaluating its validity on at least three stable compounds (DWNT-C, DWNT-C-dop-OW-Co, and DWNT-C-dop-OW-Cu) using the ZINDO/S approach yielded results listed in Table 2. These findings demonstrate its efficacy, particularly in accurately predicting transition energies to higher excited states compared to the TD-DFT.

In contrast to previous studies focusing on graphene-based nanostructures and phenine nanotubes^56–58^, our paper delves into the optical properties of single-walled carbon nanotubes (SWNTs), double-walled carbon nanotubes (DWNTs), and their derivatives. While the former investigations primarily concentrate on chemical modifications and variations in size, our study provides a comprehensive examination of the influence of dopants on the optical properties of carbon nanotubes. Through a detailed analysis of UV–Vis absorption spectra, we elucidate the intricate interplay between dopants and the optical characteristics of these nanostructures. This exploration not only enhances our understanding of the fundamental optical behaviors of carbon nanotubes but also holds significant implications for a myriad of applications ranging from photoluminescence enhancement to the development of advanced optoelectronic devices. By broadening the scope of our investigation to encompass dopant-induced modifications, our study contributes to the holistic understanding of the tunability and potential applications of carbon nanotubes in nanomaterial science and technology.

### Hydrogen storage

**Figure 5:**
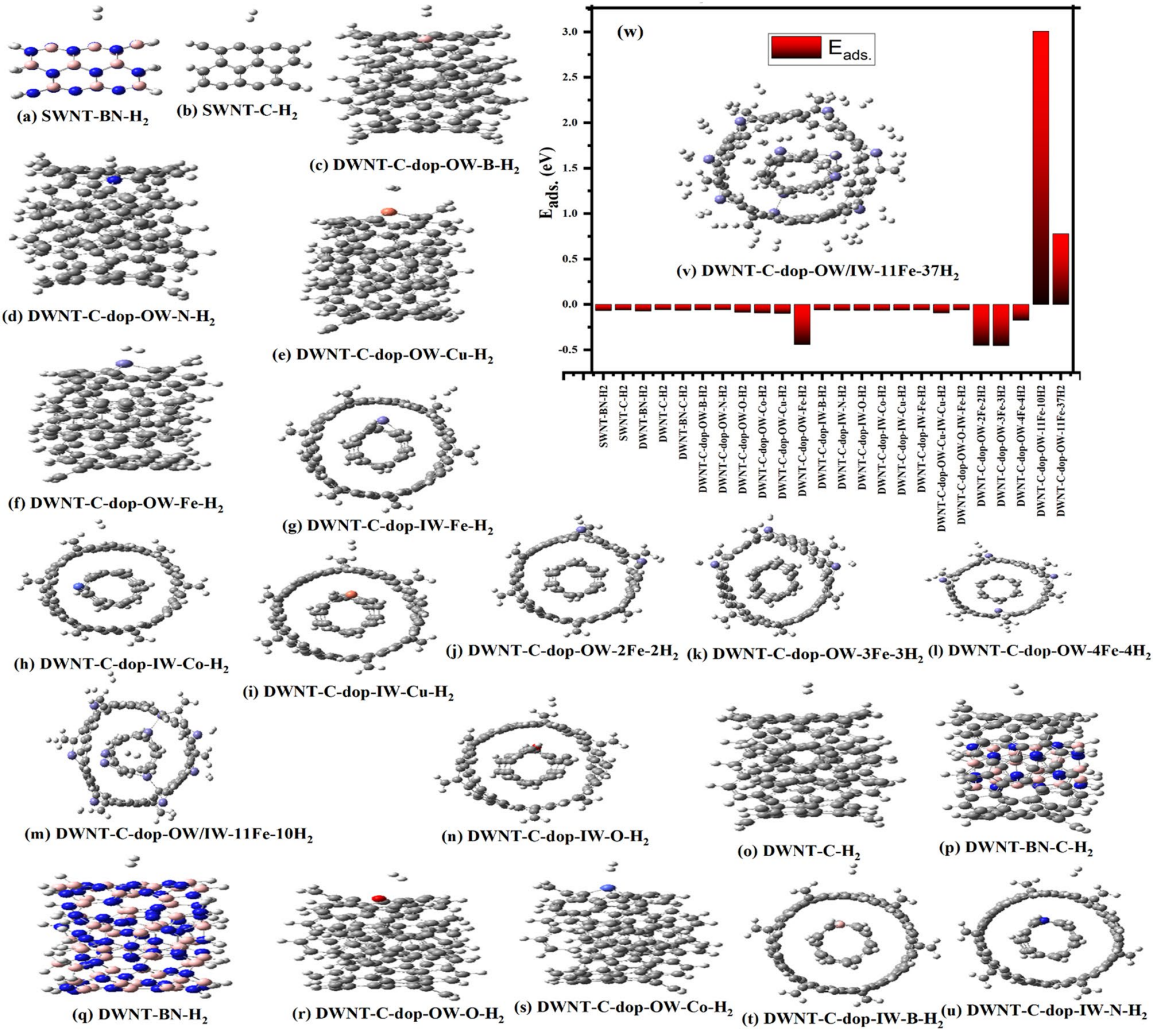
The optimized structure of SWNT-BN, SWNT-C, DWNT-BN, DWNT-C, and doped DWNT-C variants absorbing H_2_ molecule at different positions (**u**, **v**). The adsorption of one H_2_ molecule on DWNT-C doped with Fe (**g**), and adsorption of 2H_2_, 3H2, 4H2, 10H2, and 37 H2 molecules by 2Fe, 3Fe, 4Fe, 10Fe, and 11 Fe atoms (**j**–**m**, **v**) respectively. The adsorption energy diagram is shown in (**w**).

In this section, we investigate the potential utility of BN and C nanotubes for hydrogen storage, focusing on the calculation of hydrogen adsorption energy (E_ads_). The adsorption energy per H_2_ molecule (E_ads_) is determined using the formula:^34^ E_ads_ = (E_(NT-nH2)_—(E_NT_ + nE_H2_))/n, where E_(NT-nH2)_, E_NT_, and nE_H2_ represent the ground state energies of NT/H_2_, NT, and H_2_, respectively, and n is the number of adsorbed H_2_ molecules. Figure 5 (a-v) illustrates the optimized structures of SWNT-BN, SWNT-C, DWNT-BN, DWNT-C, and various doped DWNT-C variants adsorbing H_2_ molecules at different positions, offering visual insights into the diverse interactions between nanotube structures and hydrogen molecules. Notably, the adsorption configurations encompass scenarios such as a single H_2_ molecule on Fe-doped DWNT-C (panels f and g) and the adsorption of multiple H_2_ molecules by varying Fe dopant concentrations (panels j–m and v). The corresponding adsorption energy diagram (panel w) visually represents the adsorption energies for these different configurations, providing a critical visual aid to complement the comprehensive discussion on hydrogen storage capabilities, dopant influence, and the transition from negative to positive adsorption energies in the manuscript. Table 2 presents a detailed overview of the adsorption energy (E_ads_) values for various nanotube configurations, shedding light on hydrogen storage capabilities and the influence of different dopants. For SWNTs, both SWNT-BN-H_2_ and SWNT-C-H_2_ exhibit negative E_ads_ values of − 0.0672 eV and − 0.0608 eV, respectively, indicating favorable hydrogen adsorption. Moving to double-walled nanotubes (DWNTs), DWNT-BN-H_2_ and DWNT-C-H_2_ also display negative Eads values of − 0.0712 eV and − 0.0573 eV, emphasizing their potential for hydrogen storage. Introducing dopants, including N, O, B, Co, Cu, and Fe, to DWNT-C reveals nuanced impacts on E_ads_. Notably, specific dopants contribute to enhanced hydrogen adsorption, exemplified by DWNT-C-dop-OW-Fe-H_2_ with a significantly improved Eads of − 0.4405 eV. Examining the effect of Fe dopant concentration unveils interesting trends, with configurations like DWNT-C-dop-OW-2Fe-2H_2_ and DWNT-C-dop-OW-3Fe-3H_2_ exhibiting notable increases in E_ads_. However, DWNT-C-dop-OW-4Fe-4H_2_ experiences a lowering in E_ads_ (− 0.1743 eV), suggesting potential saturation or an optimal dopant concentration. Moreover, simultaneous Fe doping to both outer and inner walls in DWNT-C-dop-OW/IW-11Fe-10H_2_ yields a remarkable positive E_ads_ of 3.0075 eV, highlighting the synergistic effect of dopant distribution. This positive trend is maintained in DWNT-C-dop-OW/IW-11Fe-37H_2_ with a slightly reduced E_ads_ of 0.7763 eV. The transition from negative to positive E_ads_ values underscores the intricate interplay between dopant type, concentration, and nanotube structure, providing valuable insights for tailoring nanotube-based materials for optimal hydrogen storage.

Table 3 provides a comprehensive overview of desorption temperatures (T) for hydrogen across various nanotube configurations, including SWNT-BN, SWNT-C, DWNT-BN, DWNT-C, and doped DWNT-C variants. The desorption temperature represents the temperature at which hydrogen is released from the nanotubes. The following Van’t Hoff formula^34^ is used for the calculation of the desorption temperature (T_D_). ${T}_{D}=\left(\frac{{E}_{a}}{{K}_{B}}\right){\left(\frac{\Delta S}{R}-lnp\right)}^{-1},$ where E_a_ is the adsorption energy per H_2_ molecule, K_B_ is the Boltzmann constant, ΔS is the change in H_2_ entropy from gas to liquid phase, R is the universal gas constant, and p is the atmospheric pressure. For single-walled nanotubes (SWNTs), both SWNT-BN-H_2_ and SWNT-C-H_2_ exhibit estimated desorption temperatures of 85.84 K and 77.71 K, respectively, suggesting relatively low-temperature desorption. Transitioning to double-walled nanotubes (DWNTs), DWNT-BN-H_2_ and DWNT-C-H_2_ display desorption temperatures of 90.99 K and 73.19 K, respectively. The introduction of dopants to DWNT-C has a significant impact on desorption temperatures. Notably, specific dopants contribute to enhanced hydrogen adsorption, exemplified in DWNT-C-dop-OW-Fe-H_2_, which exhibits a substantially improved desorption temperature of 562.81 K. Simultaneous doping of Fe to both outer and inner walls in DWNT-C-dop-OW/IW-11Fe-10 H_2_ yields an extraordinary desorption temperature of 3842.83 K. This exceptionally high desorption temperature indicates a robust and stable interaction between hydrogen and the nanotube structure, suggesting promising applications in high-temperature hydrogen release.

**Table 3:** The adsorption energy (E_ads_), and the desorption temperature (T) of hydrogen on SWNT-BN, SWNT-C, DWNT-BN, DWNT-C, and doped DWNT-C variants.

Compounds	E_ads_ (eV)	T(K)	Compounds	E_ads_ (eV)	T(K)
SWNT-BN-H_2_	− 0.0672	85.84	DWNT-C-dop-IW-N- H_2_	− 0.0645	82.37
SWNT-C- H_2_	− 0.0608	77.71	DWNT-C-dop-IW-O- H_2_	− 0.0651	83.13
DWNT-BN- H_2_	− 0.0712	90.99	DWNT-C-dop-IW-Co- H_2_	− 0.0652	83.34
DWNT-C- H_2_	− 0.0573	73.19	DWNT-C-dop-IW-Cu- H_2_	− 0.0618	78.93
DWNT-BN-C- H_2_	− 0.0641	81.88	DWNT-C-dop-IW-Fe- H_2_	− 0.0610	77.99
DWNT-C-dop-OW-B- H_2_	− 0.0599	76.49	DWNT-C-dop-OW-Cu-IW-Cu- H_2_	− 0.0934	119.33
DWNT-C-dop-OW-N- H_2_	− 0.0589	75.28	DWNT-C-dop-OW-O-IW-Fe- H_2_	− 0.0603	77.05
DWNT-C-dop-OW-O- H_2_	− 0.0862	110.15	DWNT-C-dop-OW-2Fe-2 H_2_	− 0.4505	575.64
DWNT-C-dop-OW-Co- H_2_	− 0.0931	119.01	DWNT-C-dop-OW-3Fe-3 H_2_	− 0.4534	579.39
DWNT-C-dop-OW-Cu- H_2_	− 0.0980	125.24	DWNT-C-dop-OW-4Fe-4 H_2_	− 0.1743	222.71
DWNT-C-dop-OW-Fe- H_2_	− 0.4405	562.81	DWNT-C-dop-OW/IW-11Fe-10 H_2_	3.0075	3842.83

In comparing the desorption temperatures of hydrogen on pristine and doped TiO_2_ nanotubes^34^ with doped DWNT-C variants and hBNt^59^, notable differences arise. While pristine TiO_2_ nanotubes exhibit relatively low desorption temperatures ranging from 51 to 192 K, doping with elements like carbon and silicon significantly elevates these temperatures to highs of 6717 K and 4363 K, respectively. Similarly, doped DWNT-C variants demonstrate remarkable improvements, with exceptionally high desorption temperatures reaching 3842.83 K. In contrast, hBNt exhibits increased desorption temperatures ranging from 1399 to 279 K, surpassing even room temperature. These findings underscore the effectiveness of doping strategies in enhancing hydrogen desorption kinetics, with doped DWNT-C variants showing particular promise for high-temperature hydrogen storage applications.

## Conclusion

In conclusion, our study delves into the intricate structural details and properties of hexagonal boron nitride (BN) and carbon (C) nanotubes, encompassing both single-walled (SWNT) and double-walled (DWNT) configurations. Through systematic exploration and optimization, we unveil the stability and electronic characteristics of these nanotube structures. The introduction of dopants, strategically positioned within the nanotubes, showcases a nuanced influence on stability, charge distribution, and electronic behavior. The analysis of energy gaps and electronic transitions provides a comprehensive understanding of the diverse electronic properties, guiding potential applications in tailored electronic devices. Additionally, our investigation into optical characteristics reveals the impact of nanotube size and dopant type on absorption spectra, crucial for the design of nanotube-based materials in optoelectronics. Moreover, our exploration of hydrogen storage capabilities highlights promising adsorption energies and desorption temperatures, particularly in doped DWNT-C variants, suggesting potential breakthroughs in high-temperature hydrogen release applications. In essence, this study contributes valuable insights into the multifaceted properties of hexagonal BN and C nanotubes, opening avenues for their strategic utilization in various technological domains.

### Supplementary Information

Supplementary Figures.

## Data Availability

All data generated or analyzed during this study are included in this published article.
