# Supplementary material for: Exploring the structural, electronic, and hydrogen storage properties of hexagonal boron nitride and carbon nanotubes: insights from single-walled to doped double-walled configurations
Source: Sci Rep. 2024 Feb 29;14:4970. doi: 10.1038/s41598-024-55583-8 (PMC10904835; doi:10.1038/s41598-024-55583-8)
Supplement: Supplementary file 1 — Supplementary Figures. [file 41598_2024_55583_MOESM1_ESM.docx]

**Exploring the Structural, Electronic, and Hydrogen Storage Properties of hexagonal Boron Nitride and Carbon Nanotubes: Insights from Single-Walled to Doped Double-Walled Configurations**

**Mahmoud A. S. Sakr^1*^, Hazem Abdelsalam^2,3*^, Nahed H. Teleb^2,4^, Omar H. Abd-Elkader^5^, Qinfang Zhang^1*^**

^1^Center of Basic Science (CBS), Misr University for Science and Technology (MUST), 6th October City, Egypt

^2^School of Materials Science and Engineering, Yancheng Institute of Technology, Yancheng 224051, PR China

^3^Theoretical Physics Department, National Research Centre, El-Buhouth Str., 12622, Dokki, Giza, Egypt

^4^Electron Microscope and Thin Films Department, National Research Centre, El-Buhouth Str., 12622, Dokki, Giza, Egypt

^5^Department of Physics and Astronomy, College of Science, King Saud University, P.O. Box 2455, Riyadh 11451, Saudi Arabia


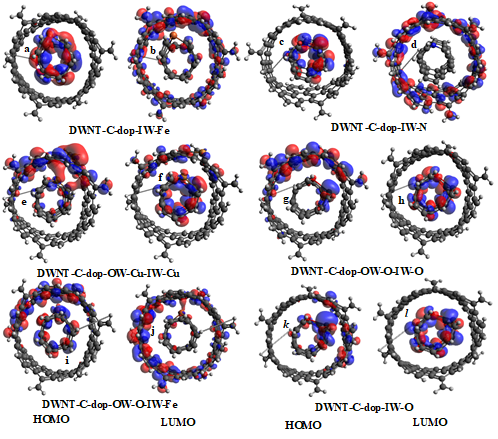


Figure S1. HOMO/LUMO MOs representations for DWNT-C-dop-IW-Fe, DWNT-C-dop-IW-N, DWNT-C-dop-OW-Cu-IW-Cu, DWNT-C-dop-OW-O-IW-O, DWNT-C-dop-OW-O-IW-Fe and DWNT-C-dop-IW-O (a-*l*).


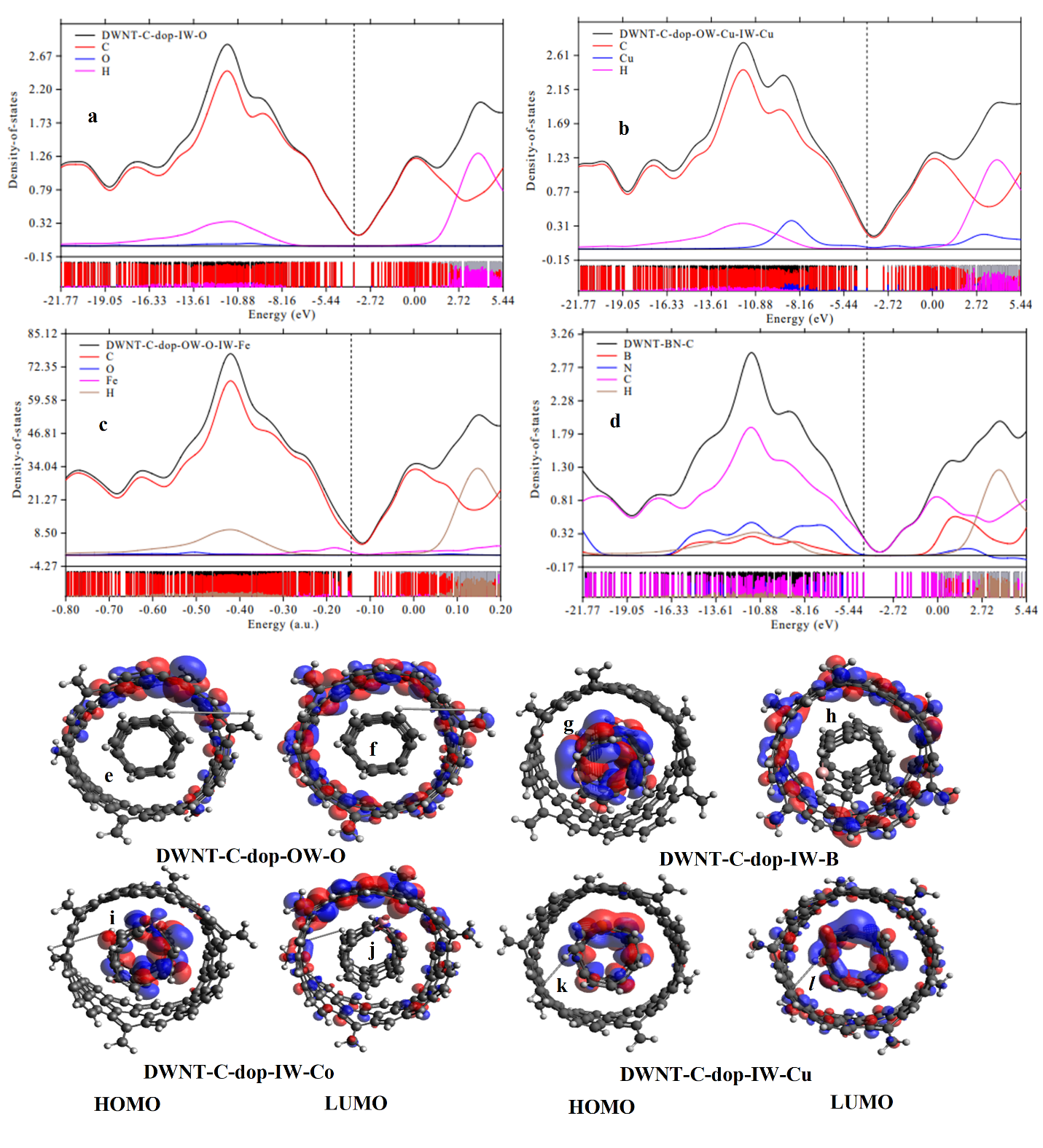


Figure S2. The partial density of states of DWNT-C-dop-IW-O, DWNT-C-dop-OW-Cu-IW-Cu, DWNT-C-dop-OW-O-IW-Fe, and DWNT-BN-C (a-d). HOMO/LUMO MOs representations for DWNT-C-dop-OW-O, DWNT-C-dop-IW-B, DWNT-C-dop-IW-Co, and DWNT-C-dop-IW-Cu (e-*l*).


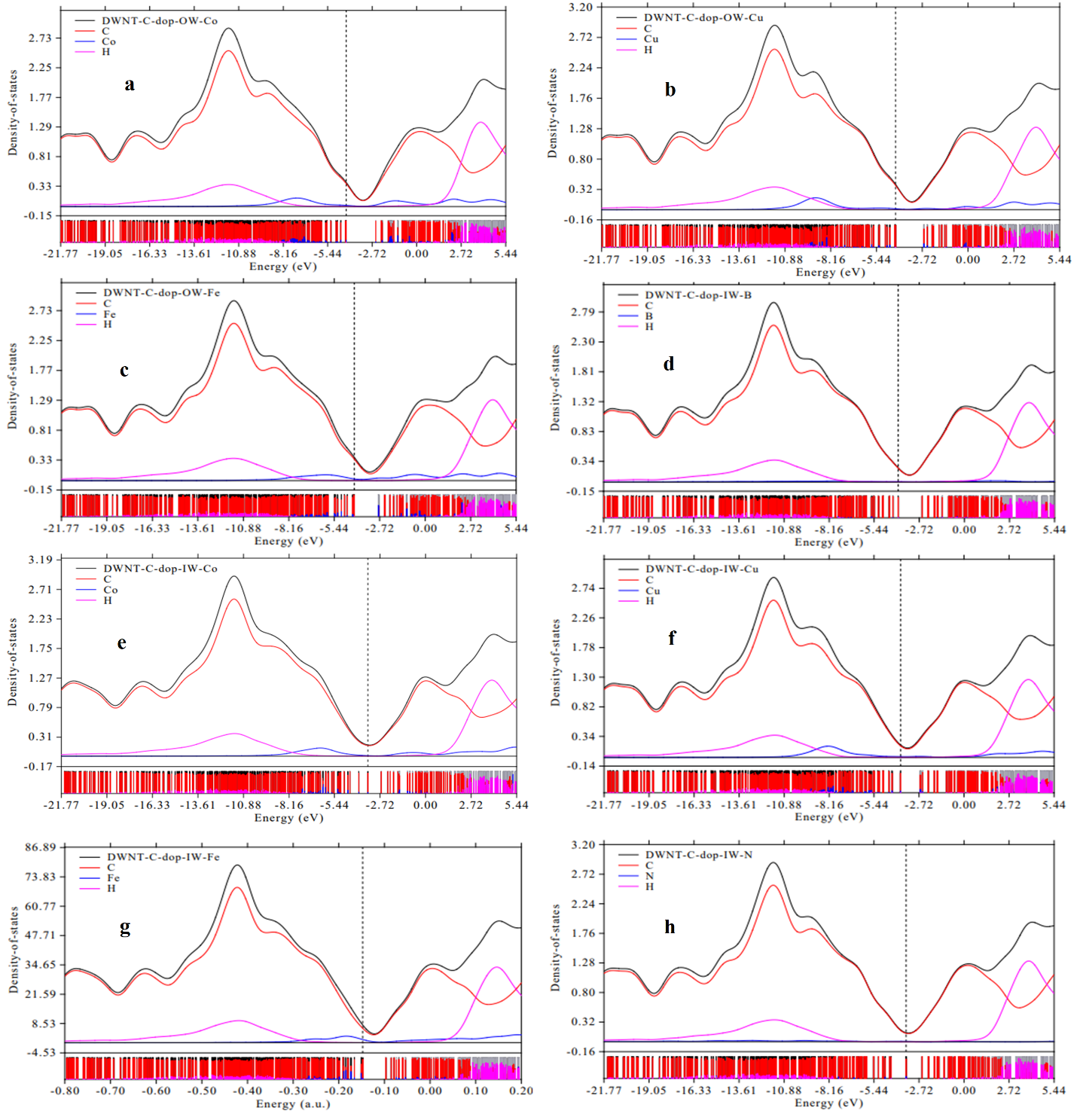


Figure S3 The partial density of states of DWNT-C-dop-OW-Co, DWNT-C-dop-OW-Cu, DWNT-C-dop-OW-Fe, DWNT-C-dop-IW-B, DWNT-C-dop-IW-Co, DWNT-C-dop-IW-Cu, DWNT-C-dop-IW-Fe, DWNT-C-dop-IW-N (a-h).
